# Microbiomes Reduce Their Host’s Sensitivity to Interspecific Interactions

**DOI:** 10.1128/mBio.02657-19

**Published:** 2020-01-21

**Authors:** Sara L. Jackrel, Kathryn C. Schmidt, Bradley J. Cardinale, Vincent J. Denef

**Affiliations:** a Department of Ecology and Evolutionary Biology, University of Michigan, Ann Arbor, Michigan, USA; b School for Environment and Sustainability, University of Michigan, Ann Arbor, Michigan, USA; c Cooperative Institute for Great Lakes Research, University of Michigan, Ann Arbor, Michigan, USA; University of Pittsburgh

**Keywords:** microbiome, eukaryotic species interactions, species coexistence, biodiversity

## Abstract

Bacteria associated with eukaryotic hosts can affect host fitness and trophic interactions between eukaryotes, but the extent to which bacteria influence the eukaryotic species interactions within trophic levels that modulate biodiversity and species coexistence is mostly unknown. Here, we used phytoplankton, which are a classic model for evaluating interactions between species, grown with and without associated bacteria to test whether the bacteria alter the strength and type of species interactions within a trophic level. We demonstrate that host-associated bacteria alter host growth rates and carrying capacity. This did not change the type but frequently changed the strength of host interspecific interactions by facilitating host growth in the presence of an established species. These findings indicate that microbiomes can regulate their host species’ interspecific interactions. As between-species interaction strength impacts their ability to coexist, our findings show that microbiomes have the potential to modulate eukaryotic species diversity and community composition.

## Abstract (precis)

Description of the Earth’s microbiota has recently undergone a phenomenal expansion that has challenged basic assumptions in many areas of biology, including hominid evolution, human gastrointestinal and neurodevelopmental disorders, and plant adaptation to climate change. By using the classic model system of freshwater phytoplankton that has been drawn upon for numerous foundational theories in ecology, we show that microbiomes, by facilitating their host population, can also influence between-species interactions among their eukaryotic hosts. Between-species interactions, including competition for resources, has been a central tenet in the field of ecology because of its implications for the diversity and composition of communities and how this in turn shapes ecosystem functioning.

## INTRODUCTION

A major control on biodiversity is mediated by how species requiring the same resources interact with each other (1, 2). For eukaryotic communities, understanding interspecific interactions is generally achieved by focusing on two focal species. The presence of a second species either shows no impact on a species’ growth rate (neutral interaction) or changes it either positively (facilitation) or negatively (competition). The type and strength of these interactions determine whether species will coexist in a community (3). Rarely has the influence of host-associated bacteria, also called microbiomes, on these interspecific interactions and, thus, on species coexistence and community composition been considered, yet microbiomes significantly influence their eukaryotic host’s fitness (4, 5) and physiology and health (6–9) and can change how the host impacts ecosystem functioning (10, 11). While these bacterial influences on their hosts are likely to have cascading effects on nontrophic interactions between hosts, explicit tests of this idea remain limited. Examples from trophic systems suggest that host-associated microbes have the potential to be key players in mediating eukaryotic interactions. For example, host-associated microbiomes mediate plant-herbivore interactions (12–14), such as the ability of pea aphids to specialize on a host plant (15). Further, host-associated microbiomes can confer resistance in aphids from parasitic wasps (16), facilitate predation activity by bioluminescent squid (17), and permit the survival of marine tube worms in nutrient-limited environments (18). Testing for the prevalence of microbiome-mediated regulation of eukaryotic interactions within trophic levels is therefore an important next step.

It has previously been shown that symbiotic bacteria and fungi can increase bioavailable nutrient pools, therefore benefiting the growth of a eukaryotic host and reducing competition between host individuals (19, 20). By measuring the growth of a small number of individual plants as a proxy for reproductive fitness, researchers have found that symbiotic fungi alter competitive interactions between two species of grassland plants (21, 22). However, natural systems are comprised of populations, not individuals, and measures of reproductive fitness inherently require multigenerational studies. Thus, it has not yet been determined how host-associated bacteria influence multigenerational, population-level ecological interactions between their hosts.

Here, we report the results of a set of experiments that show how host microbiomes alter interspecific interactions between their phytoplankton host populations. Eukaryotic phytoplankton have served as a classic model system for the development of community ecology theory ever since G. E. Hutchinson posed the paradox of the plankton (23–26). Phytoplankton are used as a model system because they have rapid generation times that allow one to characterize the population dynamics needed to quantify species interactions (3, 27–29). In addition, phytoplankton are ecologically important. They are responsible for half of Earth’s net primary productivity (30), and their community composition is a regulator of many biogeochemical cycles and food web dynamics (31, 32). Bacteria associated with phytoplankton are known to affect host fitness either negatively or positively (33–39), and these interactions can depend on highly specific chemical signaling (39). These impacts on host fitness can, in turn, alter interactions between hosts if bacteria compete for or facilitate resource capture by their phytoplankton hosts (40), affect host growth rates (41), change the mortality of either the host or the competing species (42, 43), or control the growth of parasites (44).

To explicitly test whether microbiomes alter host interspecific interactions, we used four species of algae that have previously been shown to have ecological interactions ranging from competition to facilitation (45). We evaluated all six pairwise species interactions in the presence versus the absence of phytoplankton-associated bacteria using the mutual invasibility criterion to measure the type and strength of the interspecific interaction (3, 46). This criterion uses an experimental approach in which species A and B are grown alone in monoculture and population densities are sampled through time to estimate per capita growth rates (μ*_i_*). Species A is then introduced at a low density into a culture of species B that has been grown to steady state, and vice versa. The sensitivity of each species to the interaction (*S_i_*) is calculated as the proportional increase or decrease in μ*_i_* when a species invades an established population of a second species (μ*_i_*_,invading_) relative to the μ*_i_* when the species is grown alone in monoculture (μ*_i_*_,alone_), S*_i_* = (μ*_i_*_,alone_ − μ*_i_*_,invading_)/μ*_i_*_,alone_. This criterion is a standard method for measuring the strength of competition (3, 46), but it is also more broadly suited for evaluating interspecific interactions, because significant positive or negative values of *S_i_* indicate that the established species has significant fitness effects on the invading species. Using whole bacterial communities that consisted in part of bacterial taxa with verified host-specific effects on phytoplankton growth, we show that whole bacterial communities modify the strength of phytoplankton interactions. While this did not change the type of interactions between the eukaryotic hosts or the likelihood that they could coexist, the substantial changes in host sensitivity to the presence of another phytoplankton species warrants a broader investigation into the effects of the microbiome on eukaryotic community diversity and composition.

## RESULTS

**FIG 1:**
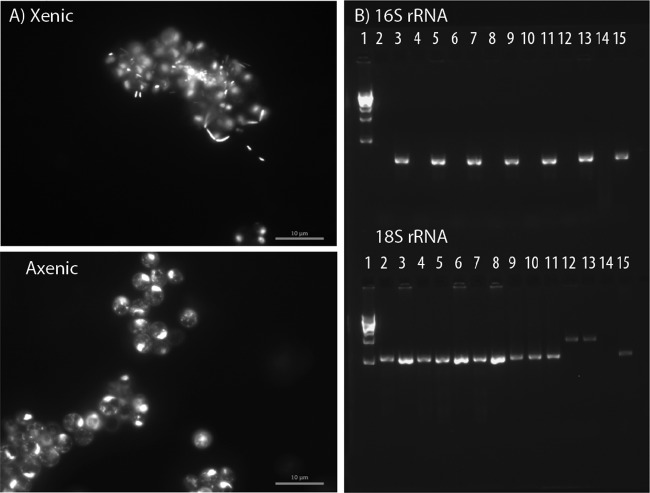
(A) Micrographs depicting the presence and absence of phytoplankton-associated bacteria prior to and after using our axenification protocol on Chlorella sorokiniana. Samples were stained with DAPI (4′,6-diamidino-2-phenylindole) and viewed at a ×100 magnification with an oil immersion lens on a Zeiss AxioImager M2 epifluorescence microscope. Bacteria and phytoplankton were visualized under a DAPI filter (bandpass, 450- to 490-nm excitation; long pass, 515-nm emission). See micrographs of the other phytoplankton species in Fig. S1 in the supplemental material. (B) Gel electrophoresis analysis of xenic and axenic phytoplankton cultures, with 16S rRNA gene amplification on the top and 18S rRNA gene amplification on the bottom. Lanes: 1, 1-kb ladder; 2 and 3, axenic and xenic Scenedesmus acuminatus, respectively; 4 and 5, axenic and xenic Coelastrum microporum, respectively; 6 and 7, axenic and xenic Monoraphidium minutum, respectively; 8 and 9, axenic and xenic Oocystis polymorpha, respectively; 10 and 11, axenic and xenic Chlorella sorokiniana, respectively; 12 and 13, axenic and xenic Selenastrum capricornutum, respectively. Negative template controls (lane 14) and a positive template control of extracted DNA from quagga mussel (Dreissena bugensis) and its associated bacteria (lane 15) were also included.

We used a single-cell sorting approach to render laboratory cultures of four species of eukaryotic green algae (Coelastrum microporum, Monoraphidium minutum, Scenedesmus acuminatus, and Selenastrum capricornutum) free of their associated bacteria (axenic). We confirmed that the algal cultures were axenic using a combination of microscopy, attempted heterotroph isolation on R2A agar (73), and colony PCR using bacterium-specific primers (Fig. 1; see also Fig. S1 in the supplemental material). We added whole bacterial communities, which had been gently dissociated and separated from xenic (bacteria present) phytoplankton, to the axenic version of the same species. This approach controls for any effects of the axenification protocol (e.g., inadvertent selection of a specific genotype of the host due to single-cell sorting). We used this culture-independent approach to retain maximal bacterial diversity by capturing both bacteria that we could isolate (Table S1) and bacteria that we were unable to culture in isolation.

10.1128/mBio.02657-19.1 FIG S1 Micrographs depicting the presence and absence of phytoplankton-associated bacteria prior to and after using our axenification protocol. All samples were stained with DAPI (4′,6-diamidino-2-phenylindole) stain and viewed at a ×100 magnification with an oil immersion lens on a Zeiss AxioImager M2 epifluorescence microscope. Bacteria and phytoplankton were visualized under a DAPI filter (bandpass, 450- to 490 nm excitation; long pass, 515-nm emission). Differences between xenic and axenic detection of fluorescence inside of algae are apparent, with nuclei and putative organelle nucleic acids being more visible in the axenic cultures. This visible variation in the signal may be attributed to stain concentrations relative to nucleic acid material and emission saturation. For example, axenic cultures would have a greater proportion of stain emitted from intracellular nucleic material, whereas in xenic cultures, the emission signal is distributed across bacterial nucleic material, resulting in a relative decreased intracellular emission from phytoplankton nucleic acids. Download FIG S1, PDF file, 0.3 MB. Copyright © 2020 Jackrel et al. 2020 Jackrel et al. This content is distributed under the terms of the Creative Commons Attribution 4.0 International license.

10.1128/mBio.02657-19.7 TABLE S1 Descriptions of all phytoplankton-associated bacterial isolates. All isolates were obtained by streaking phytoplankton cultures on solid R2A medium. Full 16S rRNA gene sequences can be found on this paper’s github page (https://github.com/sjackrel/Microbiomes-Reduce-their-Host-s-Sensitivity-to-Interspecific-Interactions). Download Table S1, PDF file, 0.1 MB. Copyright © 2020 Jackrel et al. 2020 Jackrel et al. This content is distributed under the terms of the Creative Commons Attribution 4.0 International license.

**FIG 2:**
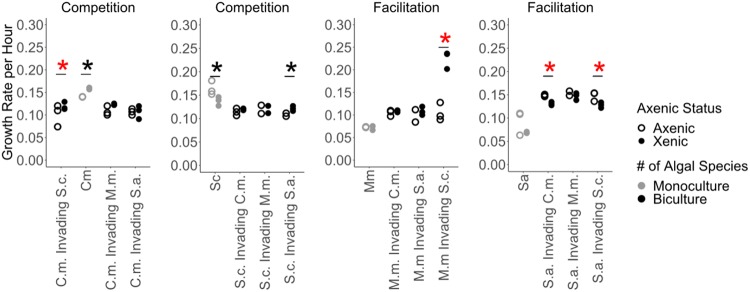
Host-associated bacterial communities alter the strength but not the type of ecological interactions between hosts. Ecological interactions between phytoplankton species ranged from competitive interactions (decreased growth rate relative to that of monoculture) to facilitative interactions (increased growth rate compared to that of monoculture). From left to right, growth rates are for Coelastrum microporum (*C.m.*), Selenastrum capricornutum (*S.c.*), Monoraphidium minutum (*M.m.*), and Scenedesmus acuminatus (*S.a.*). Phytoplankton-associated bacterial communities frequently altered the rate of exponential growth of their phytoplankton host during the first 26 h postinoculation both in monocultures and when introduced at a low density into (i.e., invading) an established phytoplankton culture (biculture) via linear regression. In addition to this model incorporating all species combinations, asterisks indicate for which species combinations the axenic status significantly affected the growth rate, as determined using two-sample *t* tests on subsets of the data. A red asterisk indicates a *P* value of <0.05, and a black asterisk indicates a *P* value of <0.10.

We showed that the microbiomes never changed the type but frequently altered the strength of host interspecific interactions. These results ranged from negative to positive ecological interactions, as in the absence of bacteria, two species of phytoplankton experienced competition when introduced as the invader and two species experienced facilitation (Fig. 2). The presence of bacteria changed the rate of per capita population growth of their hosts in monoculture as well as when the host invaded an established culture of another species, but there was a significant interaction between axenic/xenic status and species combination (Fig. 2) (in the linear model across all species combinations, *F*_1,57_ = 8.2 and *P* = 0.006 for axenic/xenic status, *F*_15,57_ = 28.6 and *P* < 0.001 for species combination, and *F*_15,57_ = 13.0 and *P* < 0.001 for interaction). Due to this significant interaction term, we tested each species combination as to whether the axenic/xenic status significantly altered the growth rate using two-sample *t* tests. Considering that four species were used in this experiment, our study tested 6 pairwise combinations with bidirectional invasion, resulting in a total of 12 combinations. Four of these 12 combinations were significant with *P* values of <0.05, and an additional three combinations showed a weak trend with *P* values of <0.10 (Fig. 2).

**FIG 3:**
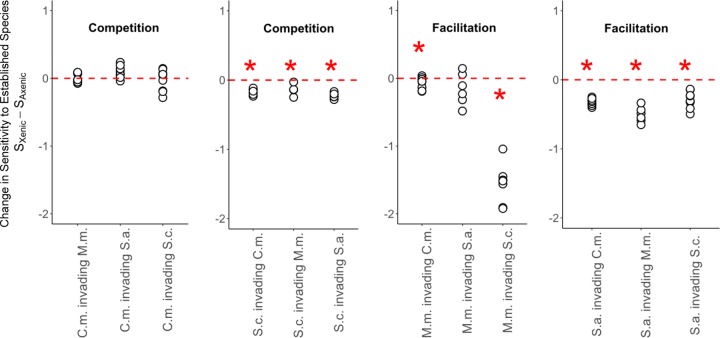
Host-associated bacterial communities reduce their host’s sensitivity to interspecific interactions. Using a layout that is parallel to that used in Fig. 2, we showed that host-associated bacteria had either no effect on interactions between their hosts or a facilitative effect on the growth of the host when rare by decreasing the rare phytoplankton host’s sensitivity to interspecific interactions. The *y* axis shows the difference between each host’s sensitivity to interaction in the xenic versus axenic treatment, where sensitivity (S*_i_*) is equal to (μ*_i,_*_alone_ − μ*_i,invading_*)/μ*_i_*_,alone_. The axenic status significantly affected the *S_i_* values according to our linear model, which was run on all four panels of data using the original *S_i_* values of xenic and axenic cultures rather than the subtracted values. In addition to this model incorporating all species combinations, asterisks indicate for which species combinations the axenic status significantly (*P* < 0.05) affected sensitivity to the established species, as determined using one-sample *t* tests on subsets of the data.

We then evaluated how the presence of bacteria altered the sensitivity of the phytoplankton hosts to the interaction with each other (*S_i_*). Increased *S_i_* values would indicate an increased magnitude of competition due to bacterial presence, while a decrease would indicate increased facilitation. Bacteria changed the algal *S_i_* in 8 out of 12 invasion experiments (Fig. 3) (one-sample *t* tests, *P* < 0.05). They did so by facilitating the growth of the rare invading species of phytoplankton to a larger degree than expected from bacterial facilitation of algal growth in monocultures, which reduced the *S_i_* of their host to interspecific interactions (Fig. 3) (in the linear model across all species combinations, *F*_11,43_ = 125.9 and *P* < 0.001 for species combination, *F*_1,43_ = 101.7 and *P* < 0.001 for axenic/xenic status, and *F*_11,43_ = 22.3 and *P* < 0.001 for interaction). In the other four invasion experiments, we found that the bacterial communities had no significant effect on the *S_i_* of the host species (Fig. 3).

For the one species pair with consistently positive *S_i_* values (C. microporum and S. capricornutum), we could then predict the impacts of bacteria on host coexistence. There have been calls to expand existing ecological theory, which currently uses equations that are valid only when *S_i_* values are >0 (47). Until coexistence theory is further developed, the availability of a single case limits our ability to generalize, but it does provide an opportunity to evaluate the degree to which bacteria can impact the probability of host species coexistence. Theory identifies two forces behind coexistence: relative fitness differences (RFDs), which give rise to growth inequalities that set up competitive hierarchies among species, and niche differences (NDs), which offset competitive hierarchies by giving species a growth advantage when rare, which serves to minimize any effect of competitive inequalities on a species’ population growth (3). Reduced sensitivity to interaction was evident in the xenic treatment relative to axenic treatment of S. capricornutum invading C. microporum, but not the inverse. This resulted in host-associated bacteria significantly increasing NDs from 0.72 ± 0.03 (mean ± standard error [SE]) within the axenic treatment to 0.82 ± 0.006 within the xenic treatment (analysis of variance [ANOVA], *F*_1,16_ = 14.51 and *P* = 0.0015). However, host-associated bacteria had no effect on RFDs (mean ± SE, 1.71 ± 0.14 for axenic treatment and 1.98 ± 0.14 for xenic treatment; ANOVA, *F*_1,16_ = 1.94 and *P* = 0.182) or predictions of competitive exclusion versus coexistence, as defined by Narwani et al. (46), where RFD must be greater than versus less than 1/(1 − ND), respectively.

In addition to our primary experiments that evaluated the effects of whole microbiomes on eukaryotic interactions, we showed that bacteria isolated from these laboratory phytoplankton cultures had host-specific impacts on the algal growth rate and carrying capacity (Fig. S5 and S6). While we cannot assess whether the isolates were reflective of the symbionts sustaining algal growth in nature, when forced into symbiosis in the lab environment, populations of algae inoculated with a bacterial isolate obtained significantly higher population densities in 62.5% of the 48 pairwise experiments between 8 bacterial isolates and 6 algal hosts (all *P* values were <0.05 by ANOVA; Fig. S5).

10.1128/mBio.02657-19.5 FIG S5 Individual isolates of phytoplankton-derived bacteria frequently alter the growth patterns of their phytoplankton hosts. Download FIG S5, PDF file, 0.3 MB. Copyright © 2020 Jackrel et al. 2020 Jackrel et al. This content is distributed under the terms of the Creative Commons Attribution 4.0 International license.

## DISCUSSION

The increased attention over the past decade to how Earth’s microbiota interacts with eukaryotes has challenged basic assumptions in many areas of biology, including hominid evolution (48), plant adaptation to climate change (4, 49), and human health (50, 51). Our results add to this list by showing that host microbiomes can alter measures of phytoplankton species coexistence that have previously been considered a fundamental property of the host species (3, 46). In addition to their inherent ecological importance, freshwater phytoplankton are also one of the few tractable study systems for quantifying the driving forces of species coexistence. Therefore, as a classic model system used in formulating numerous foundational theories in ecology that have been upheld in many other systems, our findings may pertain more broadly to how incorporating host microbiomes may alter our understanding of the maintenance of biodiversity and species coexistence. Although mutualistic microbes have previously been shown to reduce competition between plants, these studies have been limited to interactions between individuals and may not accurately predict population dynamics in a multigenerational context (20–22). Studies that have used more complex communities in a multigenerational context have tested effects on productivity and community composition, but not interaction strength (19, 52). By assessing multigenerational population dynamics, we showed that host microbiomes are frequently key in determining the strength of ecological interactions and have the potential to determine the type of these interactions.

From an applied perspective, phytoplankton species are commonly used as a system for biofuel production (53). Whereas most work has focused on improving single species performance, ecological engineering using mixed phytoplankton cultures has proven to be a fruitful avenue of research (54–57). Our research suggests that the management of associated bacteria may provide an avenue to improve algal biofuel yields when using a multispecies approach, especially as bacteria associated with microalgal cells have already been shown to increase algal carbon fixation (58).

We found that the phytoplankton microbiomes that we used have the potential to mediate host interactions; however, there are limitations to our study and critical future steps that are necessary to determine the broad relevance of these findings in nature. First, we relied on two-species interactions that form the foundation of community ecology theory but that may not adequately predict dynamics in more diverse communities (59). Our study did not focus on revising modern coexistence theory but, rather, provided an empirical test using the existing theory. Our data therefore provide a new perspective that host-associated microbes play an important role in affecting measures of species interactions that are currently widely accepted (3).

A second set of concerns reflects on the nature of the microbiomes used in our experiments, as lab-based phytoplankton cultures, and particularly their microbiomes, may not reflect their natural counterparts. To rationalize the use of this lab-based model system for understanding natural community dynamics, we confirmed through background experiments that bacterial isolates from these cultures are not merely coresiding with phytoplankton but have significant effects on phytoplankton population growth (Fig. S5 and S6). These effects of the bacteria associated with our lab cultures on phytoplankton growth are in line with observations from other studies that used freshly isolated phytoplankton and their associated bacteria from nature (35, 36, 38, 39), thus supporting the validity of our lab-based model system for understanding natural community dynamics. However, we did not establish the life history of all bacterial taxa associated with phytoplankton, and further study is necessary to show the extent to which these associations are intentional and persistent. For example, certain taxa have been shown to be persistently associated with phytoplankton (60, 61), but a broader understanding of the temporal and spatial stability of these phytoplankton-associated microbial communities is essential. Furthermore, our reintroduction of whole bacterial communities undoubtedly added both bacteria closely associated with algae and those free-living within the algal media. As we did not distinguish these two groups in our experiments, we cannot make conclusions regarding the mechanisms that underpin how bacteria alter algal interactions in our experiments, nor can we make conclusions whether the mutually beneficial interactions that we observed (i.e., algae support bacterial growth, bacteria promote algal growth) reflect a more generic effect of nutrient cycling between functional groups (autotroph-heterotroph) or a species-specific symbiosis between algae and phycosphere bacteria. However, this boundary between free-living and closely associated phycosphere bacteria appears to be more fluid than previously thought. Recent evidence from video microscopy of diatom cells has shown the phycosphere to be a highly dynamic environment of motile taxa responding rapidly via chemotaxis to gradients in phytoplankton exudates, effectively transitioning between closely associated and free-living status (71). Given this nature of the phycosphere and our use of dense algal cultures in media with no carbon source beyond bicarbonate and algal exudates, most “free-living” taxa were dependent on an algal host.

10.1128/mBio.02657-19.6 FIG S6 Host-associated bacteria alter host population dynamics. When added to axenic phytoplankton hosts, most bacterial isolates derived from phytoplankton microbiomes altered the host growth rate and carrying capacity. Effects were both host and bacterial symbiont dependent. Logistic growth curves were fit to chlorophyll *a* fluorescence-based estimates of phytoplankton population densities to obtain estimates of steady-state density (*K*) and the exponential rate of growth (μ). Ratios of *K* and μ for xenic relative to axenic phytoplankton were calculated: values of >1 indicate that the bacterial isolate increased the measure relative to that under the axenic condition. Download FIG S6, PDF file, 0.1 MB. Copyright © 2020 Jackrel et al. 2020 Jackrel et al. This content is distributed under the terms of the Creative Commons Attribution 4.0 International license.

It must also be recognized that all phytoplankton in nature have associated bacteria and that interactions without bacteria do not occur. The goal of this study was to determine how much the associated bacteria change the interactions between hosts as they would occur based on host traits alone. A critical next step to determine the extent to which phytoplankton microbiomes regulate phytoplankton community ecology is to test whether impacts on host species interactions are variable depending on the composition of the phytoplankton microbiomes. Further studies could clarify by what mechanisms the microbiome changes eukaryotic interactions. For example, testing whether these interactions are affected by the occurrence of bacterial taxa known to regulate nutrient dynamics, such as the N-fixing *Rhizobiaceae* (i.e., isolate no. 3, *Rhizobium* sp.), would be a valuable future direction. In addition, the factors that determine microbiome composition need to be further determined. Host genotype has been shown to influence the microbiome composition among genotypes of phytoplankton and plant species (63–66), though the relative importance of host and environment still needs further quantification.

In conclusion, considering the current rapid losses of Earth’s biodiversity, with about 1 million species of plants and animals being at risk of extinction (67), it is important to understand all controls over species interactions and diversity. The ecological interactions between species that underpin coexistence and thus diversity have been mostly determined by considering only traits expressed by the host species. While it could be argued that the microbiome could be categorized as an environmental factor, unlike other environmental factors (e.g., nutrient and light levels, predator and herbivore pressures), the microbiome has the potential to alter traits beyond the limits of the host’s genetics (e.g., associated bacteria fixing atmospheric nitrogen for their host, microbial communities mitigating drought stress, or viruses adding gene content to increase rates of photosynthesis) (4, 76, 77). Further, unlike other environmental factors, microbiomes may in part be transmitted between generations (64, 68), though the heritability of microbiomes, mostly assessed for mammalian gut microbiomes, is generally low for horizontally acquired symbionts. Our results indicate that we need to expand our view of ecological interactions between eukaryotes as being in part the consequence of traits encoded by their microbiomes. Considering the extensive reports of microbiome effects on the fitness of plants, phytoplankton, and animals (4, 5, 39), we propose that similar effects on host interspecific interactions and community composition may be widespread. How microbiomes and their impact on eukaryotic interactions and diversity will change in light of anthropogenic disturbance is unresolved, but our insights emphasize the need to further explore these questions.

## MATERIALS AND METHODS

### Phytoplankton materials.

Phytoplankton species cultures were supplied from the University of Texas Culture Collection of Algae in 2011 and were then maintained in laboratory slant cultures under a light intensity no higher than 30 μmol · m^−2^ · s^−1^ at 15°C on COMBO medium (UTEX; Austin, TX, USA). Six species of unicellular algae were used for population-level growth experiments: Coelastrum microporum, Selenastrum capricornutum, Scenedesmus acuminatus, Monoraphidium minutum, Oocystis polymorpha, and Chlorella sorokiniana. Both C. sorokiniana and O. polymorpha belong to the order Chlorellales, while the others belong to the order Sphaeropleales. All six species are unicellular algae. Our prior studies suggested that these species represent species with a range of competitive interactions, from strong competitors to facilitators (45). We then chose the first four species listed for community-level experiments based on their distinct morphological characteristics and similar rates of growth. Phytoplankton were grown on solid and liquid COMBO growth medium for all experiments and maintenance of cultures (69). All incubations were on shaker tables set to a continuous 80 rpm under a light intensity of 81 μE within a Percival chamber set to a 16-h light and 8-h dark cycle and 20°C.

### Axenification protocol.

We rendered the phytoplankton species axenic using a combined strategy of ultrasonication to liberate attached bacterial cells and single-cell fluorescence-activated cell sorting (FACS) onto solid growth media, following the general protocol established by Cho et al. (74; see also reference 70). We sonicated 20 ml of culture maintained at a density of 1 × 10^8^ cells·ml^−1^ on ice at 20 W for 30 s using a Fisher sonic dismembrator model 100. Sonication was repeated three times with 1 min of rest between sonications. Samples were then gently centrifuged at 900 × *g* for 5 min, the supernatant was discarded, and the pellet was resuspended with 20 ml COMBO medium. Flow cytometry sorting was performed using a FACS Synergy Head No. 1 cell sorter located in a biological safety cabinet to maintain sterility (University of Michigan Biomedical Research Flow Cytometry Core, Ann Arbor, MI, USA). Populations of the target species of phytoplankton were separated from the bacterial population using intrinsic cellular properties, such as morphology, internal complexity, and autofluorescence, based on forward and side scatter. Single cells were sorted into 96-well plates containing COMBO agar medium using a blue excitation laser and a peridinin chlorophyll protein broad-pass emission filter (488-nm excitation, 665/30-nm emission). Ninety-six-well plates were covered with translucent, adhesive Breathe-Easy films to allow gas exchange and minimize contamination. The plates were incubated for 7 to 12 days until growth was visible. Phytoplankton colonies formed from this single-cell sorting event were viewed under a dissecting microscope for bacterial contamination. Morphologies that appeared contaminant free (70) were streaked onto individual 100-mm petri dishes of COMBO agar and then confirmed to be axenic by the methods described below.

### Confirmation of axenic state.

The axenic status of each species was confirmed through microscopy, colony PCR, and attempted heterotroph isolation on R2A agar. Liquid cultures of phytoplankton were stained with DAPI (4′,6-diamidino-2-phenylindole) and visualized on a fluorescence microscope (Axio Imager 2 Zeiss microscope). We confirmed the absence of unculturable bacteria through colony PCR using 799mod7 (5′-GGA TTA GAT ACC CKG GT -3′) and 1392R (5′- ACG GGC GGT GTG TRC -3′) 16S rRNA gene fragment primers to minimize chloroplast amplification (75). We dissolved a single colony of phytoplankton in 10 μl nuclease-free water and incubated the solution for 10 min at 100°C using a PCR Mastercycler (nexus gradient). The product supernatant was used as the PCR template. Each 25-μl PCR mixture contained 13 μl nuclease-free water, 10 μl NEBNext High-Fidelity 2× PCR master mix, 1 μl template, 0.5 μl 10 μM forward primer, and 0.5 μl 10 μM reverse primer. PCR conditions were as follows: 94°C for 3 min, 35 cycles of denaturation (94°C for 45 s), annealing (46°C for 60 s), and extension (72°C for 90 s), and a final extension at 72°C for 10 min. The amplified PCR product was analyzed by electrophoresis on a 1% agarose gel (see Fig. S5 in the supplemental material). We confirmed the absence of culturable bacteria by dissolving a colony in 1 ml sterile COMBO medium and streaking onto R2A agar. The plates were incubated at room temperature in the dark and analyzed for growth after 2 to 5 days. Confirmation of the continued axenic status of all stock cultures and experimental replicates was confirmed by microscopy and DAPI staining.

### Mutual invasibility experiment.

Once we obtained axenic phytoplankton populations, we reintroduced whole bacterial communities into aliquots of our axenic populations. This approach controls for the effects of the axenification protocol on phytoplankton population growth, such as potentially reduced phytoplankton genetic diversity imposed by single-cell sorting. To obtain whole bacterial communities for reintroduction to axenic phytoplankton, we sonicated 20 ml of each species of xenic phytoplankton for 30 s at 20 W and passed the volume through 0.8-μm-pore-size filters twice to remove all eukaryotic cells. We then immediately added this bacterial filtrate to an aliquot of axenic phytoplankton belonging to the same species as the bacterial filtrate. For example, 0.5 ml of bacterial filtrate derived from a xenic Monoraphidium minutum population was added to 1 ml of an axenic Monoraphidium minutum population. Axenic aliquots received an additional 0.5 ml of sterile COMBO medium rather than the bacterial filtrate. All 1.5-ml xenic and axenic cultures were incubated in 12-well plates sealed with Breathe-Easy sealing membranes for 13 days. We used this 2-week period of bacterium-phytoplankton coincubation to facilitate reassociations between the host and its associated bacterial community. All phytoplankton cultures were then transferred to 100 ml of sterile COMBO medium for an additional 7 days to obtain larger volumes for the following experiment. We used this approach to add whole bacterial communities rather than only a restricted number of bacterial isolates that could be cultured or only those taxa previously known to reside within the phytoplankton microbiome. This approach likely includes both bacteria residing in the phycosphere, i.e., bacteria directly attached to and within the diffusive boundary layer around phytoplankton cells (62), and free-living bacteria residing in the medium outside of the phycosphere. Nonetheless, all of these bacteria thrive in medium where the only carbon source beyond bicarbonate is exudates of the phytoplankton host species and together provide a fitness benefit to the host species. Furthermore, the phycosphere is dynamic, with motile bacteria entering and exiting the phycosphere via rapid chemotaxis (71). Hence, we refer to all of these bacteria as “phytoplankton associated.”

We tested all pairwise combinations of each of the four phytoplankton species but paired xenic phytoplankton only against other xenic phytoplankton and axenic phytoplankton only against other axenic phytoplankton. Each pairwise combination was examined in triplicate, with all replicates being spatially randomized in a Percival incubator. We started 72 axenic and xenic phytoplankton cultures inoculated at 1,000 cells ml^−1^ into 100 ml of sterile COMBO medium in 150-ml Erlenmeyer flasks. Additionally, to monitor for steady-state density while minimizing the risk of bacterial contamination, we concurrently inoculated an additional 24 axenic and xenic flasks intended to remain as monocultures throughout the experiment. The population density within these designated monoculture flasks was monitored via chlorophyll *a* fluorescence on a BioTek plate reader. We found that all eight populations had reached steady-state population growth by 22 days postinoculation (Fig. S3). At this steady-state time point, we surveyed all axenic flasks for bacterial contamination via fluorescence microscopy.

10.1128/mBio.02657-19.2 FIG S2 Fluorescence-based tracking of phytoplankton population density was used to determine when the invading species could be added to a steady-state population. The invading species was added after the final time point shown (∼410 h). Download FIG S2, PDF file, 0.02 MB. Copyright © 2020 Jackrel et al. 2020 Jackrel et al. This content is distributed under the terms of the Creative Commons Attribution 4.0 International license.

10.1128/mBio.02657-19.3 FIG S3 Cell density curves for all monocultures and invaders in the mutual invasibility experiments. Cell densities were counted with a hemocytometer at five time points. All replicates within treatments are shown with best-fitting third-order polynomials, as determined with log-ratio tests, except for the S. acuminatus xenic monoculture, which was better modeled with a second-order polynomial. Decelerating growth was noticeable by the fourth time point for most treatments; therefore, maximum growth rates were determined using the first three time points. Download FIG S3, PDF file, 0.1 MB. Copyright © 2020 Jackrel et al. 2020 Jackrel et al. This content is distributed under the terms of the Creative Commons Attribution 4.0 International license.

The invading species was then inoculated at 1,000 cells ml^−1^ into the 72 flasks that had reached a steady-state density. Starting at 19 h after invasion, we preserved 55 μl of each of the 72 algal cultures in 10% phosphate-buffered formalin and repeated the preservation procedure every 12 h for 84 h. Concurrently with cell density preservation, community density was monitored by measurement of chlorophyll *a* fluorescence. We relied on fluorescence-based estimates to track approximately the densities of phytoplankton bicultures to determine when to add the second, invading culture and begin sampling for cell counts. As fluorescence can be affected by factors beyond cell density (e.g., cell size and activity), all growth rate calculations determined to assess microbiome impacts on host interactions relied on cell counts. We counted cell density in all monocultures and for each algal species per biculture using preserved samples collected at five time points (0 h, 19 h, 26 h, 74 h, and 96 h) by counting all cells within a minimum of 1.8 μl of culture using a hemocytometer.

### Population-level growth experiments.

Each of the six xenic phytoplankton strains was also used to isolate host-associated bacteria. Stock cultures were sonicated at 20 W to disassociate the bacteria and then streaked onto R2A medium, which is suitable for the isolation of aerobic and facultative heterotrophic bacteria from potable water samples. Isolate 1*_C.m._* was isolated from C. microporum, isolates 2*_M.m._* and 3*_M.m._* were isolated from M. minutum, isolates 4*_O.p._* and 5*_O.p._* were isolated from O. polymorpha, isolates 6*_S.a._* and 7*_S.a._* were isolated from S. acuminatus, and isolate 8*_S.c._* was isolated from S. capricornutum (Table S1). All isolated colonies were sequenced using the universal 16S rRNA primers 27F and 1492R. Single colonies were dissolved in 50 ml liquid R2A medium, incubated for 24 h at 23°C, and diluted to an optical density at 600 nm of approximately 1.0 (range, 0.72 to 1.16).

For population-level growth experiments, phytoplankton populations were inoculated at 5,000 cells ml^−1^ in sterile COMBO liquid medium alone or with the addition of 3.3 μl of one of the eight bacterial isolates. Treatments were incubated in triplicate in 48-well plates sealed with Breathe-Easy sealing membranes. Replicates were spatially randomized with no more than one replicate per individual well plate. Chlorophyll *a* fluorescence was tracked with a BioTek plate reader every 1 to 2 days for 25 days.

### Analysis.

For surveys of bacterial isolates on trajectories of phytoplankton growth, we fit logistic growth models to estimates of phytoplankton population density using chlorophyll *a* fluorescence measures. Models were fit to each replicate to obtain multiple, independent estimates of the carrying capacity and the exponential rate of growth for each treatment using the growthrates package in R software. Estimates of carrying capacities and exponential rates of growth within treatments were then averaged, the ratios of each bacterial treatment over the axenic treatment were calculated, and the ratios were visualized with the pheatmap package in R software. Growth curves are illustrated as the mean ± SE of the fluorescence measures across replicates. Significant differences in phytoplankton growth curves with and without bacterial isolates were determined using fluorescence data with analysis-of-variance models containing day and bacterial treatment as the fixed effects.

To determine the best-fitting models for cell density count data, we compared linear, second-order, and third-order polynomials with log-ratio tests (Fig. S3). We noted decelerating growth by the fourth time point for most treatments (Fig. S4a) but similar coefficients of variation among biological replicates within treatments across time points (Fig. S4b). To calculate the growth rates used in our mutual invasibility experiment (Fig. 2), we calculated maximum growth rates using the first three time points over the first 26 h postinoculation via the fit_easylinear function in the growthrates package in R software. For supplementary analyses showing growth rates (μ) between just two time points (Fig. S4), we used the following equation: μ = (1/*T*)·ln(*D_T_*/*D*_0_), where *T* is the time elapsed in hours between the measurements of initial cell density (*D*_0_) and final cell density (*D_T_*).

10.1128/mBio.02657-19.4 FIG S4 (A) Growth rate estimates, determined using the equation μ = (1/*T*)·ln(*D_T_*/*D*_0_), decelerated significantly after the third cell density count, which was 74 h postinoculation. Each point represents one sample, where there were 3 biological replicates of each of the 12 pairwise phytoplankton combinations, for both the xenic and axenic bacterial treatments. (B) The coefficient of variation of cell counts was similar across all time points. Each point represents one coefficient of variation measure for each of the 12 pairwise phytoplankton combinations for both the xenic and axenic treatments. For the data shown in both panels, the phytoplankton cell density was determined using a hemocytometer to count the number of cells within 1.8 μl. For higher-density samples, we counted hemocytometer grids until at least 200 cells were counted. Given the growth rate declines but the similar coefficients of variation across time points, we used only the first two time points to estimate the rate of exponential growth. Download FIG S4, PDF file, 0.1 MB. Copyright © 2020 Jackrel et al. 2020 Jackrel et al. This content is distributed under the terms of the Creative Commons Attribution 4.0 International license.

Niche differences were quantified as the geometric mean of the *S_i_*s, which is $\text{ND}=1-\sqrt{S_{\text{A}}\cdot S_{\text{B}}}$ for two species (species A and B). RFD is the standard deviation of the geometric mean, where $\text{RFD}=\sqrt{S_{\text{A}}/S_{\text{B}}}$ for two species and where *S*_A_ is ≥*S*_B_ (72). Greater NDs reduce the sensitivity of both species to competition, whereas greater RFDs cause species to be asymmetrically affected by competition such that one species’ *S_i_* increases while the other species’ *S_i_* decreases. Freshwater plankton are one of few study systems in which we know how to separate the influence of ND and RFD on species interactions and coexistence, specifically by quantifying sensitivities using the mutual invasibility approach (46, 72). Increasing ND and decreasing RFD increase the probability of coexistence; however, NDs have been shown to be the more important driver in explaining coexistence (46).

### Data availability.

All data and R scripts are available on this paper’s github page (https://github.com/sjackrel/Microbiomes-Reduce-their-Host-s-Sensitivity-to-Interspecific-Interactions).
