# Supplementary material for: Microbiomes Reduce Their Host’s Sensitivity to Interspecific Interactions
Source: mBio. 2020 Jan 21;11(1):e02657-19. doi: 10.1128/mBio.02657-19 (PMC6974562; doi:10.1128/mBio.02657-19)

Fig. S1. Micrographs depicting presence and absence of phytoplankton-associated bacteria prior to and after using our axenification protocol. All samples were stained with DAPI (4’,6-diamidino-2-phenylindole) stain and viewed at 100x magnification with oil-immersion on a Zeiss AxioImager M2 epifluorescence microscope. Bacteria and phytoplankton were visualized under a DAPI filter (BP 450 - 490 nm excitation, LongPass 515 nm emission). Differences between xenic and axenic detection of fluorescence inside of algae is apparent, with nuclei and putatively organelle nucleic acids being more visible in the axenic cultures. This visible variation in signal may be attributed to stain concentrations relative to nucleic acid material and emission saturation. For example, axenic cultures would have a greater proportion of stain emitted from intracellular nucleic material, whereas, in xenic cultures the emission signal is distributed across bacterial nucleic material resulting in a relative decreased intracellular emission from phytoplankton nucleic acids.

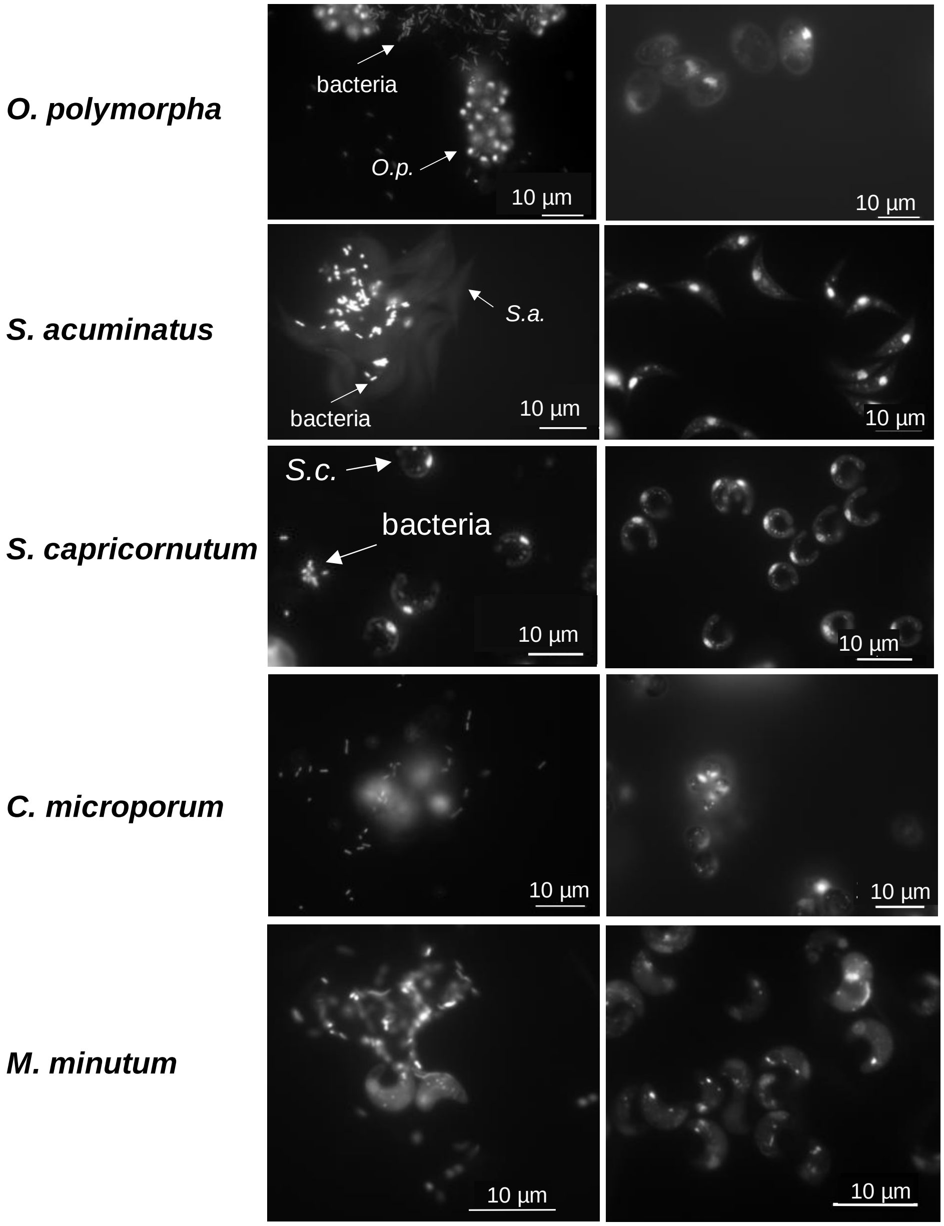

Supplement: FIG S1 [file mBio.02657-19-sf001.pdf]
