# Supplementary material for: Microbiomes Reduce Their Host’s Sensitivity to Interspecific Interactions
Source: mBio. 2020 Jan 21;11(1):e02657-19. doi: 10.1128/mBio.02657-19 (PMC6974562; doi:10.1128/mBio.02657-19)
Supplement: TABLE S1 [file mBio.02657-19-st001.pdf]

Table S1. Descriptions of all phytoplankton-associated bacterial isolates. All isolates were obtained by streaking phytoplankton cultures on solid R2A media. Full 16S sequences can be found on this paper’s github page: <https://github.com/sjackrel/Microbiomes-Reduce-their-Host-s-Sensitivity-to-Interspecific-Interactions>.

Isolate #	Algal Host	Morphological Description	Identification
$1_{C.m.}$	*C. microporum*	Large, orange	*Blastomonas natatoria*
$2_{M.m.}$	*M. minutum*	Dark orange	Unknown
$3_{M.m.}$	*M. minutum*	Small, clear, white	*Rhizobium sp.*
$4_{O.p.}$	*O. polymorpha*	Light orange	*Microbacterium sp.*
$5_{O.p}$	*O. polymorpha*	Darker orange, smaller	Uncultured bacteria
$6_{S.a.}$	*S. acummatus*	Small, yellow, translucent	*Microbacterium sp.*
$7_{S.a.}$	*S. acummatus*	Orange	*Sphingomonas sp.* or *Blastomonas natatoria*
$8_{S.c.}$	*S. capricornutum*	Orange	*Mycobacterium cosmeticum*
