# Supplementary material for: Microbiomes Reduce Their Host’s Sensitivity to Interspecific Interactions
Source: mBio. 2020 Jan 21;11(1):e02657-19. doi: 10.1128/mBio.02657-19 (PMC6974562; doi:10.1128/mBio.02657-19)

Fig. S5. Individual isolates of phytoplankton-derived bacteria frequently alter growth patterns of their phytoplankton hosts.

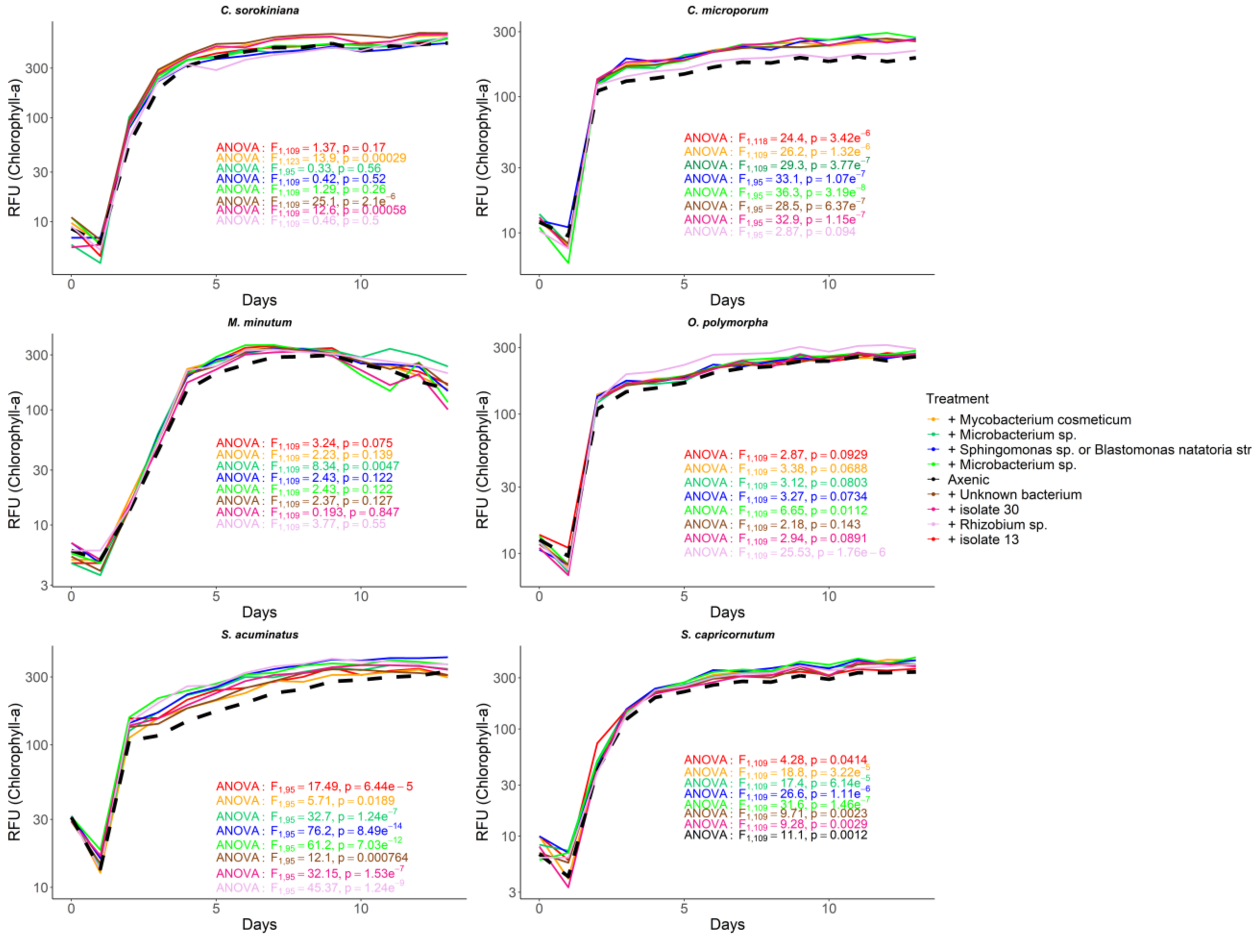

Supplement: FIG S5 [file mBio.02657-19-sf005.pdf]
