# Supplementary material for: Microbiomes Reduce Their Host’s Sensitivity to Interspecific Interactions
Source: mBio. 2020 Jan 21;11(1):e02657-19. doi: 10.1128/mBio.02657-19 (PMC6974562; doi:10.1128/mBio.02657-19)

Fig. S6. Host-associated bacteria alter host population dynamics. When added to axenic phytoplankton hosts, most bacterial isolates derived from phytoplankton microbiomes altered host growth rate and carrying capacity. Effects were both host and bacterial symbiont-dependent. Logistic growth curves were fit to chlorophyll-a fluorescence based estimates of phytoplankton population densities to obtain estimates of steady-state density (K) and exponential rate of growth (μ). Ratios of K and μ for xenic relative to axenic phytoplankton were calculated: values > 1 indicate the bacterial isolate increased the measure relative to the axenic condition.

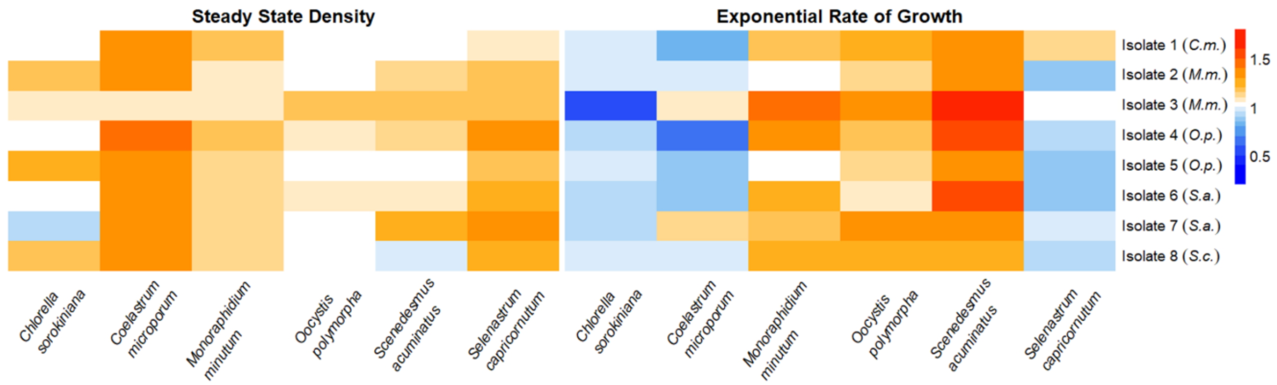

Supplement: FIG S6 [file mBio.02657-19-sf006.pdf]
