# Supplementary material for: Microbiomes Reduce Their Host’s Sensitivity to Interspecific Interactions
Source: mBio. 2020 Jan 21;11(1):e02657-19. doi: 10.1128/mBio.02657-19 (PMC6974562; doi:10.1128/mBio.02657-19)

Fig. S2. Fluorescence based tracking of phytoplankton population density was used to determine when the invading species could be added to a steady state population. The invading species was added after the final time point shown (~ 410 h).

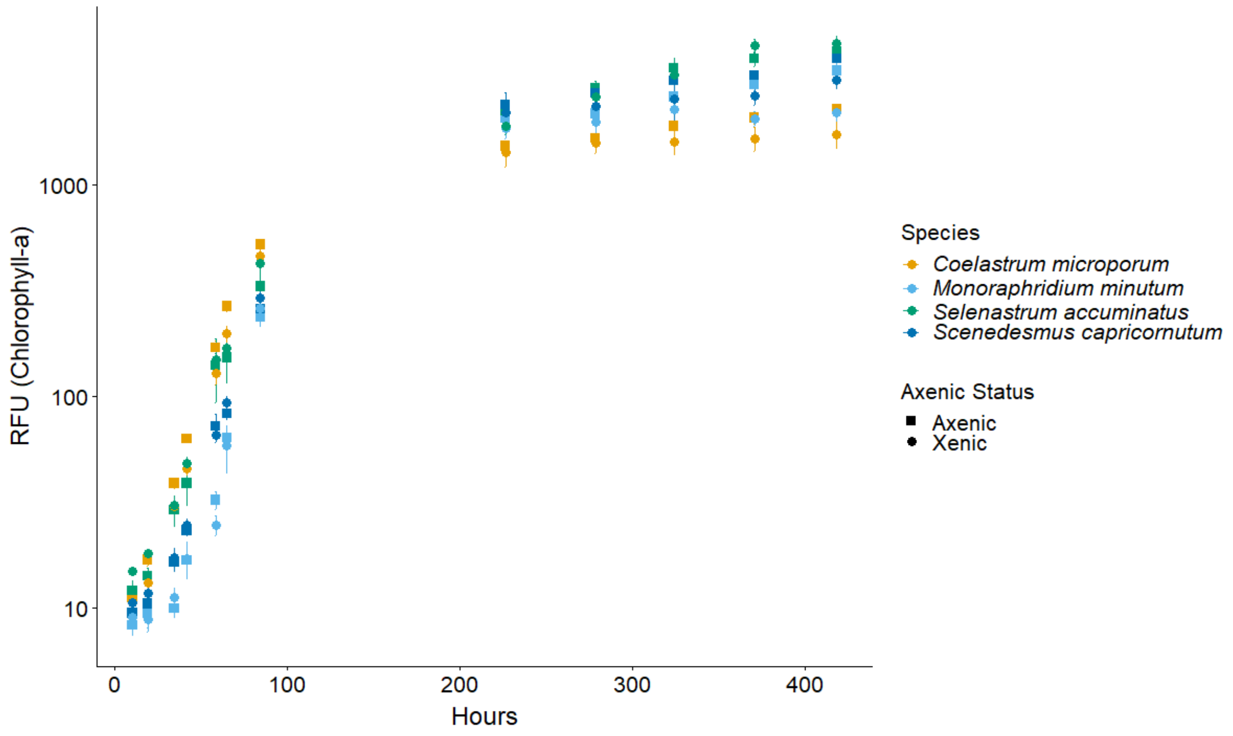

Supplement: FIG S2 [file mBio.02657-19-sf002.pdf]
