# Supplementary material for: Microbiomes Reduce Their Host’s Sensitivity to Interspecific Interactions
Source: mBio. 2020 Jan 21;11(1):e02657-19. doi: 10.1128/mBio.02657-19 (PMC6974562; doi:10.1128/mBio.02657-19)

Fig. S3. Cell density curves for all monocultures and invaders in the mutual invasibility experiments. Cell densities were counted with a hemocytometer at five time points. All replicates within treatments are shown with best fitting third order polynomials as determined with log-ratio tests, except for the *S. acuminatus* xenic monoculture which was better modeled with a second order polynomial. Decelerating growth was noticeable by the fourth time point for most treatments, therefore maximum growth rates were determined using the first three time points.

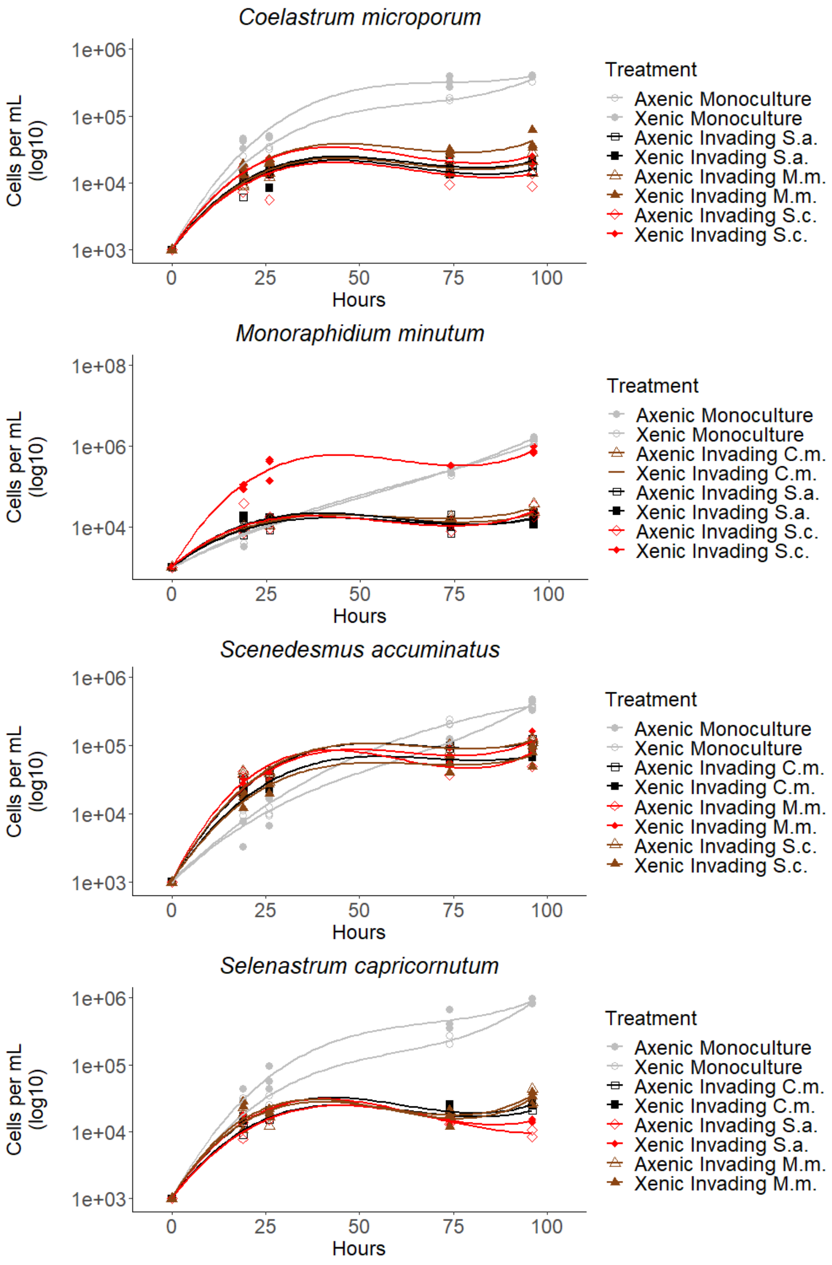

Supplement: FIG S3 [file mBio.02657-19-sf003.pdf]
