# Supplementary material for: Microbiomes Reduce Their Host’s Sensitivity to Interspecific Interactions
Source: mBio. 2020 Jan 21;11(1):e02657-19. doi: 10.1128/mBio.02657-19 (PMC6974562; doi:10.1128/mBio.02657-19)
Supplement: FIG S4 [file mBio.02657-19-sf004.pdf]

Fig. S4. A) Growth rate estimates using equation $\mu = (1/T)*\ln(D_T/D_0)$ decelerated significantly after the third cell density count, which was 74 hours post inoculation. Each point represents one sample, where there were 3 biological replicates of each of the 12 pairwise phytoplankton combinations, for both the xenic and axenic bacterial treatments. B) The coefficient of variation of cell counts was similar across all time points. Each point represents one CV measure for each of the 12 pairwise phytoplankton combinations, for both the xenic and axenic treatments. For data shown in both figure panels, phytoplankton cell density was determined using a hemocytometer to count the number of cells within 1.8 µl. For higher density samples, we counted hemocytometer grids until at least 200 cells were counted. Given growth rate declines but similar CV across time points, we used only the first two time points to estimate rate of exponential growth.

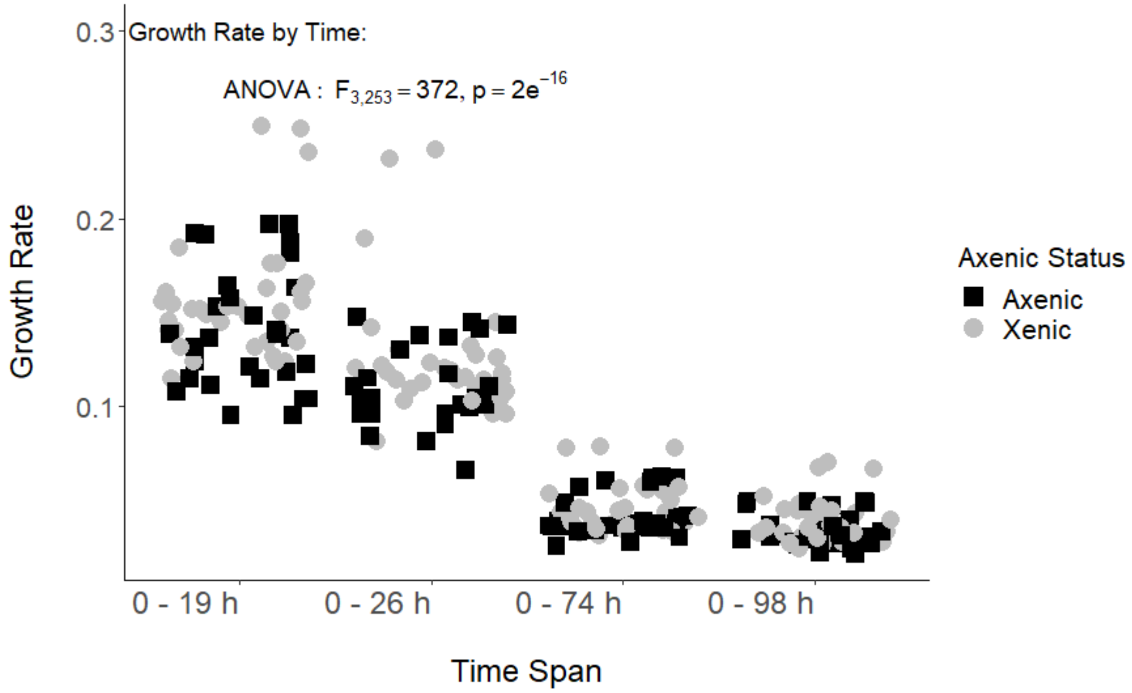

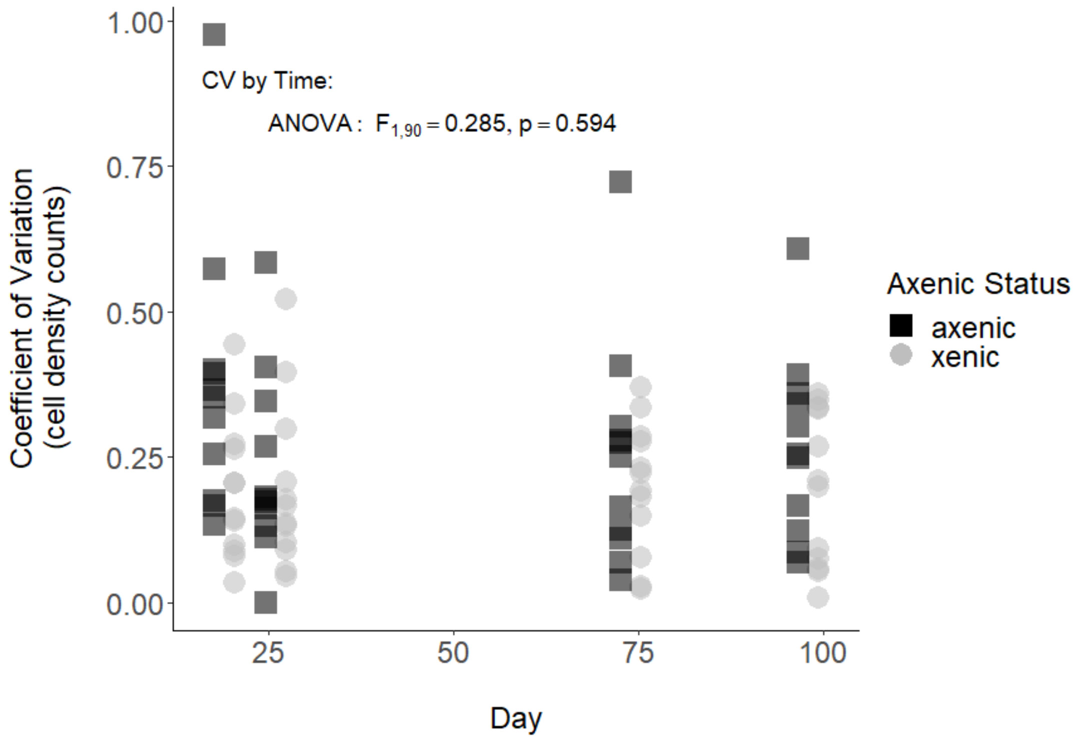

1.00
0.75
0.50
0.25
0.00
CV by Time:
ANOVA : F1,90 = 0.285, p = 0.594
Coefficient of Variation
(cell density counts)
25
50
75
100
Day
Axenic Status
axenic
xenic
